# Insights into the Mechanical Behaviour of a Layered Flexible Tactile Sensor

**DOI:** 10.3390/s151025433

**Published:** 2015-10-02

**Authors:** Julián Castellanos-Ramos, Rafael Navas-González, Iván Fernández, Fernando Vidal-Verdú

**Affiliations:** 1 Departamento de Electrónica, E.T.S.I. Informática, Universidad de Málaga, Andalucía Tech, Campus de Teatinos, 29071 Málaga, España; E-Mails: julian@elca.uma.es (J.C.-R.); rjnavas@uma.es (R.N.-G.); 2 Instituto de Investigación Biomédica de Málaga (IBIMA), 29010 Málaga, España; 3 Center for Electrochemical Technologies (CIDETEC), Paseo de Miramón, 196, 20009 Donostia-San Sebastián, España; E-Mail: ifernandez@cidetec.es

**Keywords:** tactile sensor, screen-printing technology, conductive polymers

## Abstract

This paper shows realizations of a piezoresistive tactile sensor with a low cost screen-printing technology. A few samples were fabricated for different materials used as insulator between the conductive layers and as top layer or cover. Both can be used to tune the sensitivity of the sensor. However, a large influence is also observed of the roughness at the contact interface on the sensitivity and linearity of the output, as well as on mismatching between the outputs from different taxels. The roughness at the contact interface is behind the transduction principle of the sensor, but it also limits its performance if the wavelength of the roughness is comparable or even longer than the size of the contacts. The paper shows experimental results that confirm this relationship and discusses its consequences in sensor response related to the materials chosen for the insulator and the cover. Moreover, simulations with FEA tools and with simple models are used to support the discussions and conclusions obtained from the experimental data. This provides insights into the sensor behaviour that are shared by other sensors based on the same principle.

## 1. Introduction

Several principles have been exploited to build tactile sensors (basically arrays of force sensing units or *taxels*) [1], resulting, mainly, in two large sets of sensors: piezoresistive and capacitive. The requirements of a large area, and also flexibility, impose severe limitations on the technology that can be used to make these sensors. One possible direct approach is to build a large array of force sensors made of silicon on a flexible printed circuit, though the cost can be high. MEMS on polymers [2] overcome the limitations of silicon in terms of brittleness and lack of flexibility, though they have not yet led to a mature enough to be commercialised. Moreover, the advantage of such an approach, in terms of accuracy and resolution in force measurement, is often handicapped by the need for an outer cover that avoids damage to the sensors and improves the impedance characteristics of the skin at the contact interface. For instance, if a rubber or elastomer is used as the sensor’s outer layer, the physical properties of the rubber will affect the sensor reading. As a consequence, since the rubber has the ability to store energy, undesired sources of errors arise, such as hysteresis and drift.

Therefore, despite other cheaper technologies being more error prone than silicon based ones, the final result is not notably worse in terms of performance. For instance, a large set of capacitive sensors based on flexible electrodes, fabric and elastomers has been developed and is in the marketplace for diverse applications [3], and a few have been specifically built for robotics [4,5].

Other large sets of tactile sensors are based on piezoresistive principles, *i.e.* the conductivity between two electrodes depends on the pressure on the taxel. They are basically composed of a layer of sensitive material placed on or between an array of electrodes. There are many examples of realizations that follow this basic approach, though they do not share the same working principle. Some realizations are based on conductive rubbers, where conductive paths are created because the concentration of conductive particles increases as the pressure increases, as stated by the percolation theory [6,7,8,9]. Others are based on quantum tunneling effects [10]. The resistance decreases when the load increases in these cases, while it increases with the load in the reported rubber nanocomposite in [11], due to the destruction of conductive paths created by contact or tunneling effects by transverse slippage of conductive black carbon particles. All these proposals are based on a change of the volume of the piece of sensitive material.

Other realizations take advantage of the change of the contact area at the microscopic scale due to the roughness at the contact interface, thus achieving thinner sensors [12,13,14]. Many sensors based on this approach are commercially available [15,16,17] and have been used in robotics [18,19,20]. The change of the conductivity of these materials is used to tune the sensitivity of the sensor. A large spatial resolution is achieved by the arrays in [14,15,17], although their output actually depends on the compliance of the object in contact, so this should be taken into account when they are calibrated. The reason is that they are arrays of sensitive points surrounded by a non-sensitive area. If the object in contact has low compliance, the force is concentrated at the sensitive points and the output is large. On the contrary, if the object in contact is soft, the output is smaller [21]. Other sensors do not suffer from this limitation, at least not to the same extent, since the active area is the whole contact interface between the sensitive material and the electrodes, and this area is that of the taxel. This is the case of the commercial sensors from Weiss robotics [22], where the sensors are composed of the sensitive material atop of an array of electrodes on a flexible printed circuit board [23]. The sensitivity and range of the sensor is determined by the properties of the sensitive material. The authors have reported sensors made with a similar technology, and have shown that the sensitivity can be tuned with the conductivity of the polymer at the contact interface [24].

The work presented here explores a different method that consists of changing the mechanical properties (*i.e.* the compliance) of some layers in a realization with a screen printing technology. Samples of the proposed sensor were fabricated using insulating materials of different compliance. Moreover, some pieces of different materials were used as covers or top layers. Simple calculations and FEA simulations on the ideal model predict a capability to tune the sensitivity and range of the sensor by choosing the suitable insulator and cover. However, other fundamental and practical issues that have to be taken into account arise. These are basically those related to the limited size of the contact electrodes with respect to the long wavelength features of the roughness along the contact interface. The dependence of the conductance of these factors has been studied for a long time in tribology [25]. Recent works [26,27] conclude that these features clearly determine the conductance at the interface in terms of sensitivity or linearity. This obviously affects the performance of tactile sensors based on piezoresistive materials on arrays of electrodes. This is the case with the sensors in this paper and many experimental measurements confirm it. Discussions based on simulations and other simple models explain this behaviour and provide conclusions to assess sensor performance depending on the materials used as insulator and cover.

Though focused on a specific technology, several discussions made in this paper are applicable to many sensors made with conductive layers on electrodes. Its importance in the realizations based on the roughness at the contact interface is obvious, but it also has influence in the other cases based on changes in volume if they also have rough contact interfaces. It can only be neglected when the active layer is glued to the electrodes with a conductive adhesive [28,29], but it should be taken into account if both layers are not glued but are merely in contact. A relevant work shows this fact in [30], where pressure sensitive materials are tested with contact interfaces glued or not glued to the electrodes with a conductive adhesive. The result is quite surprising since the sensitivity to pressure is mainly due to the rough contact interface and not to the change of volume in the cases explored. Therefore, we presume that these effects should be taken into account in all cases with rough contact interfaces.

The content of the paper is organized as follows: Section 2 introduces the design and realization of the sensor with a screen-printing technology. Section 3 describes the experimental setup and instrumentation used to obtain some relevant data of the materials and the fabricated sensors, and to measure their output. Section 4 provides the ideal model and detailed discussions about its limitations, and proposes another, still simple but extended model, that is able to better explain the sensor’s behaviour. Section 5 shows the experimental results and their related discussion on the basis of the analysis and models given in Section 4. Finally, Section 6 summarizes the main conclusions obtained from previous discussions.

## 2. Design and Realization

**Figure 1:**
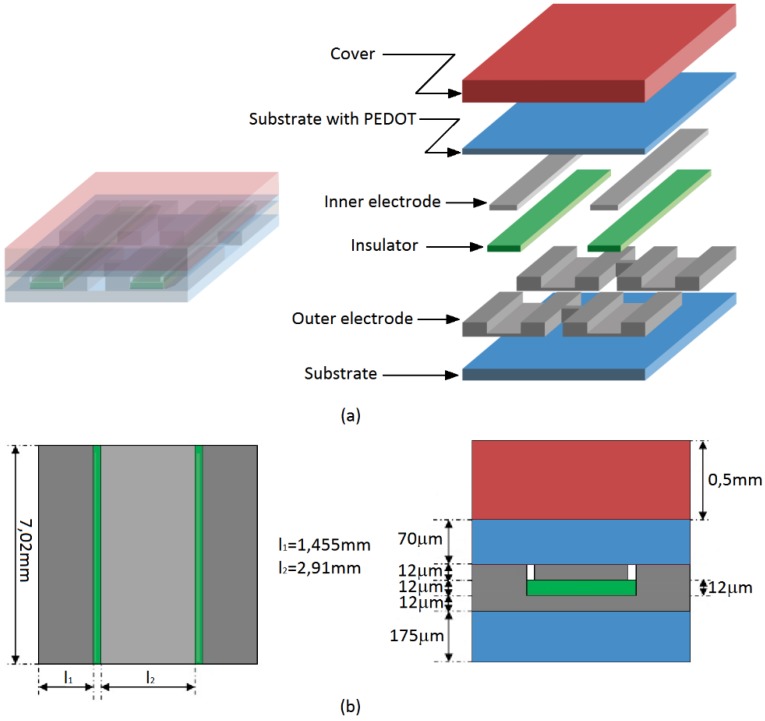
(**a**) 3D scheme and connection of the taxels in the array. (**b**) Proposed design of the taxel.

Figure 1 depicts the sensor that has been proposed and tested in this paper. It is composed of six layers which are from bottom to top: the substrate (PET), the outer electrode, the insulator, the inner electrode, the polymer ink based (PEDOT) on plastic (PET), and the cover. Similar design conditions for both interfaces with the inner and outer electrodes are established this time. First, both are placed at the same height to assure that both electrodes are in contact with the conductive polymer at very low pressures and avoid a pressure threshold in the sensor response [24]. Second, the contact area between the electrodes and the sensitive material is a key factor to determine the sensor sensitivity. The larger this area is, the larger the sensitivity of the sensor is. We are usually interested in as high a spatial resolution as possible, so this means the area is reduced as much as possible. Moreover, the area at both contact interfaces, between the sensitive material and the inner electrode and between the sensitive material and the outer electrode, must be the same to achieve maximum sensitivity [24].

**Figure 2:**
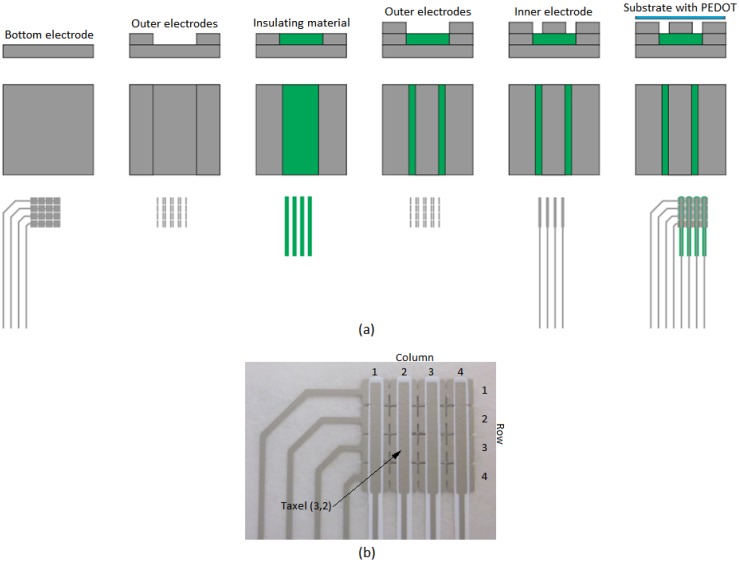
(**a**) Manufacturing process steps and (**b**) photograph of one sensor.

Six 4 × 4 sensors were built to carry out the experiments of this paper. They differ in the material used as insulator. The fabrication process was carried out with a semiautomatic shuttle table screen printing machine (Thieme 1010 E, Teningen, Gernamy) [31] with moving print table for printing on rigid and flexible materials, as used in electronic applications. Figure 2a shows the manufacturing process steps:

1. Initially, a silver conductive layer (bottom electrode) is deposited onto the 175 µm PET (polyethylene terephthalate) flexible plastic support and cured at 130 °C for 4 min in a natural convection oven (Carbolite PN 200).
2. Another conductive layer is placed atop the bottom electrode, and cured again in the oven at 130 °C for 4 min.
3. The insulating material is printed over it. The insulating materials have different thermal curing profiles.
4. Another conductive layer is placed atop the bottom electrode, and cured again in the oven at 130 °C for 4 min (outer electrode).
5. In the last screen printing step, the inner conductive electrode is deposited on the insulating material to reach the same height as the outer electrode.
6. In the final step, a film of conductive polymer PEDOT is deposited by spin-coating on a 70 µm thick layer of PET. This layer is placed on top of the previous one with the PEDOT in contact with the electrodes.

The printing of the layers in steps 1 to 5 is made through masks. Figure 2b shows a photograph of a sensor with row and column indexes to identify the taxels in the array.

## 3. Experimental Setup

### 3.1. Setup to Test the Tactile Sensors

**Figure 3:**
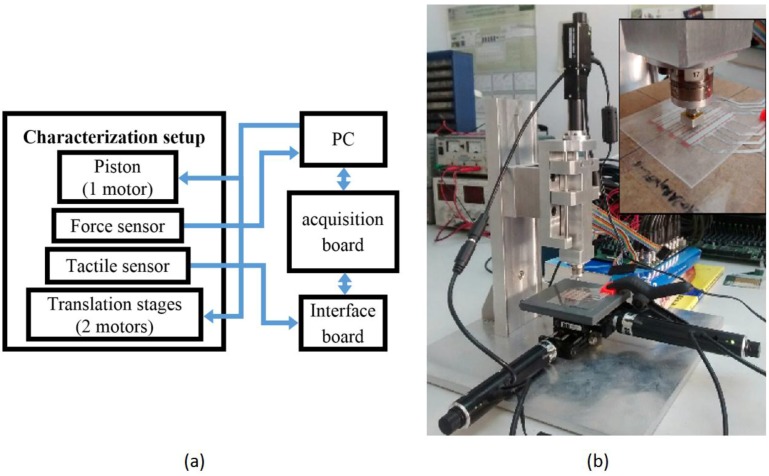
Characterization setup: (**a**) block diagram and (**b**) photograph.

A block diagram of the setup used to obtain the results of this paper is depicted in Figure 3a, and Figure 3b shows a photograph of it. It is composed of a translation stage with three micro-step motors. One of them (T-NA08A50 from Zaber, Vancouver, BC, Canada) controls a piston with a spring inside that exerts the force in *z* axis while the others (T-LA60A from Zaber) move the stage along *x* and *y* axes. A precision force sensor (nano17 from ATI Industrial Automation, Apex, NC, USA) is placed at the end of the piston to register the force exerted against the tactile sensor. The motors and the nano17 sensor have their own control and acquisition electronics and are connected to a computer. The nano17 sensor is able to measure normal forces up to 70 N with a resolution of 1/80 N and has a 5 mm diameter circular probe. The T-NA08A50 motor provides a maximum operating load of 50 N, and the T-LA60A motors provide a maximum of 15 N. An interface board was developed to scan and provide the output voltage for every taxel in the tactile sensor. These voltages are registered by a signal acquisition board (USB-6259 BNC by National Instruments Spain S.L., Las Rozas, Madrid, Spain) and sent to the PC via USB. An application was also developed on Labview™ to control the whole system.

### 3.2. Young’s Modulus Estimation

Two different procedures were used to estimate Young’s Modulus, a microhardness measurement system (FISCHERSCOPE HM2000, Windsor, CT, USA) with a Vickers’ pyramid indenter and a tensile test with a testing machine from INSTRON (Norwood, MA, USA). The indentation test was used to estimate the modulus of the thin layers such as the insulators and the electrodes while the tensile tests were used to estimate the modulus of the remaining layers, *i.e.* the covers and the PET. Table 1 shows the values of the estimated Young’s modulus.

**Table 1:** Young’s Modulus values.

Layer	Young’s Modulus (Pa)
Insulator WhiteUV	2.3 × 10^9^
Insulator Green	1.53 × 10^9^
Insulator GreenBlue	1.5 × 10^9^
Insulator RedEL	0.1 × 10^9^
Insulator Blue	1.7 × 10^9^
Insulator TranspUV	3.3 × 10^9^
Cover Pt	0.14 × 10^6^
Cover Red	0.68 × 10^6^
Cover Transp	323.52 × 10^6^
Cover PC	1299 × 10^6^
Substrate (PET)	2704.87 × 10^6^
Electrode	2.96 × 10^9^

### 3.3. Profilometries

During the fabrication process, the profilometries of the printed layers were registered with a Vaccaro’s Form Talysurf Intra profilometer (Leicester, UK) [32]. Some of them are shown later (see Section 4.4) as a resource to explain the sensor behaviour.

## 4. Analysis and Modelling of the Sensor Static Response

### 4.1. Basic Electrical Model

**Figure 4:**
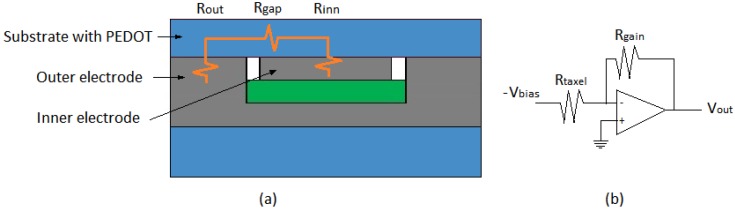
(**a**) Electrical model of the taxel. (**b**) Readout circuitry.

Figure 4a shows the electrical model of a taxel in the tactile sensor, where *R_out_* and *R_inn_* are the resistances associated to the contact interface between the sensitive layer with the conductive polymer and the outer and inner electrodes, respectively.

*R_gap_* models the resistance of the conductive polymer in the gap between both electrodes. The voltage output provided by the common signal conditioning circuit for piezoresistive tactile sensors in Figure 4b is given by the expression [24]:

(1) $$V_{out}=\frac{R_{gain}}{R_{taxel}(P)}\cdot V_{bias}$$

where $R_{taxel}(P)$ is a function of the pressure exerted on the taxel given by:

(2) $$R_{taxel}(P)=R_{inn}(P)+R_{out}(P)+R_{gap}$$

The dependence of $R_{taxel}(P)$ on *P* is conditioned by the microscopic roughness of the conducting sheet in the side that makes contact with the electrodes in the described technology [33].

Classical results on tribology establish a linear relationship between total normal force on two electrodes in contact and the resulting electrical conductance between them, and assume an elastic behaviour [34] and non-adhesive contact. Other more recent models confirm this linear relationship, although only if the distribution of contact sizes and local pressures remains constant over a wide range of loads [26,27,35]. Therefore, under this assumption we can write:

(3) $$C_{x}=k_{x}\cdot P_{x}$$

where the index *x* refers to the electrode (inn or out) and $k_{x}$ is a constant that depends on the electrical and mechanical properties of the materials in contact [33]. The resulting conductance of the taxel from Equations (2) and (3) is:

(4) $$C_{taxel}(P)=C_{inn}(P_{inn})||C_{out}(P_{out})||C_{gap}$$

where:

(5) $$(a||b)=\frac{a\cdot b}{a+b}$$

*C_gap_* is large (the conductivity of the polymer is 8.2 S/m) compared to the others and has little dependence on *P* [30], especially in the sensor of this paper whose sensitive material is a thin film. If *C_gap_* is neglected and both inner and outer contacts are identical, we can write from Equations (1), (3) and (4):

(6) $$V_{out}=\frac{\alpha_{inn}\cdot F_{inn}\cdot \alpha_{out}\cdot F_{out}}{\alpha_{inn}\cdot F_{inn}+\alpha_{out}\cdot F_{out}}\cdot R_{gain}\cdot V_{bias}=\alpha \cdot \frac{F_{inn}\cdot F_{out}}{F_{inn}+F_{out}}\cdot R_{gain}\cdot V_{bias}$$

where $\alpha_{x}=k_{x}/A_{x}$ being $A_{x}$ the area of the electrode x, and $\alpha =\alpha_{inn}=\alpha_{out}$ if the size of both contacts is the same, as said in Section 2. $F_{inn}$ and $F_{out}$ are normal forces at the inner and outer contacts respectively. If we assume:

(7) $$F=F_{inn}+F_{out}$$

(8) $$F_{inn}=a\cdot F$$

where the parameter a∈[0,1] determines the balance of force between both electrodes and F is the total normal force on the taxel, we can write from Equations (6), (7) and (8):

(9) $$V_{out}=k\cdot a\cdot \left(1-a\right)\cdot F$$

Therefore we obtain a linear relationship between the output voltage and the normal force on the taxel with a sensitivity:

(10) S=k⋅a⋅(1−a)

Note that this sensitivity is maximum when a=0.5.

### 4.2. Basic Mechanical Model

**Figure 5:**
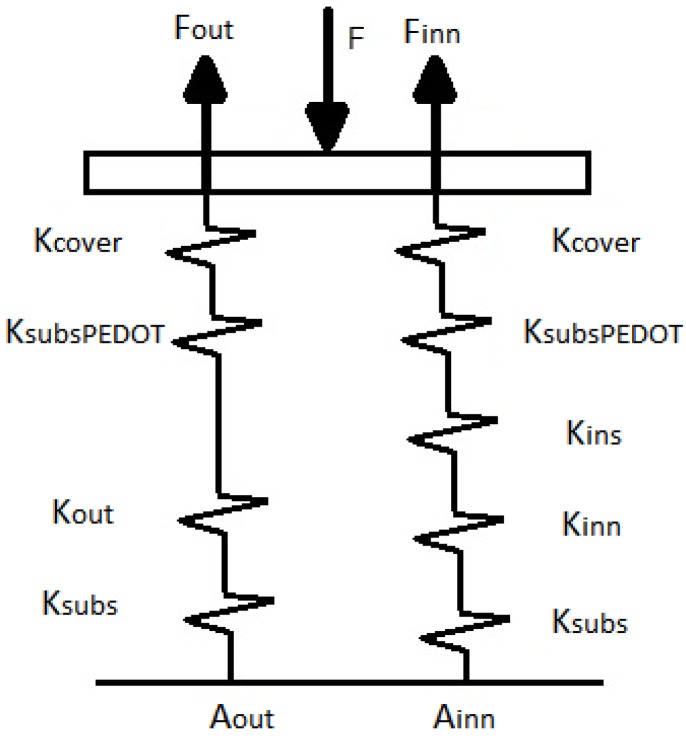
Basic mechanical model of the taxel.

Since the sensor is made of layers of continuous elastic materials a first simple model based on beams with linear elastic constants can help to understand the sensor behaviour and provide guidelines to its design [36]. This approach gives the simple model of Figure 5 for the sensor in Figure 1. The sensor is modelled as a stacked structure of layers with area $A_{layer}$, thickness $l_{layer}$ and elastic constant given by:

(11) $$K_{layer}=\frac{E_{layer}\cdot A_{layer}}{l_{layer}}$$

where $E_{layer}$ is the Young’s modulus of the layer.

From Figure 5, we can write the following expressions for the forces at the contact interfaces with the electrodes:

(12) $$\begin{array}{l} F_{inn}=\frac{\left(K_{\mathrm{cov}er}||K_{subsPEDOT}||K_{ins}||K_{inn}||K_{subs}\right)}{K_{eq}}\cdot F\\ F_{out}=\frac{\left(K_{\mathrm{cov}er}||K_{subsPEDOT}||K_{out}||K_{subs}\right)}{K_{eq}}\cdot F \end{array}$$

where $K_{eq}=\left(K_{\mathrm{cov}er}||K_{subsPEDOT}||K_{ins}||K_{inn}||K_{subs}\right)+\left(K_{\mathrm{cov}er}||K_{subsPEDOT}||K_{out}||K_{subs}\right)$ is the equivalent elastic constant of the whole taxel. From Equations (8) and (12) we can write:

(13) $$a=\frac{\left(K_{\mathrm{cov}er}||K_{subsPEDOT}||K_{ins}||K_{inn}||K_{subs}\right)}{K_{eq}}$$

Therefore, ideally it is possible to tune the sensitivity of the sensor if the geometry and mechanical properties of the layers, *i.e.* their elastic constants, are chosen properly. Note that the condition *a* = 0.5, *i.e.* the forces on both electrodes are equal and the sensitivity is maximum, only fulfilled for $K_{out}=K_{ins}||K_{inn}$ or:

(14) $$K_{ins}=\frac{K_{out}\cdot K_{inn}}{K_{inn}-K_{out}}$$

**Figure 6:**
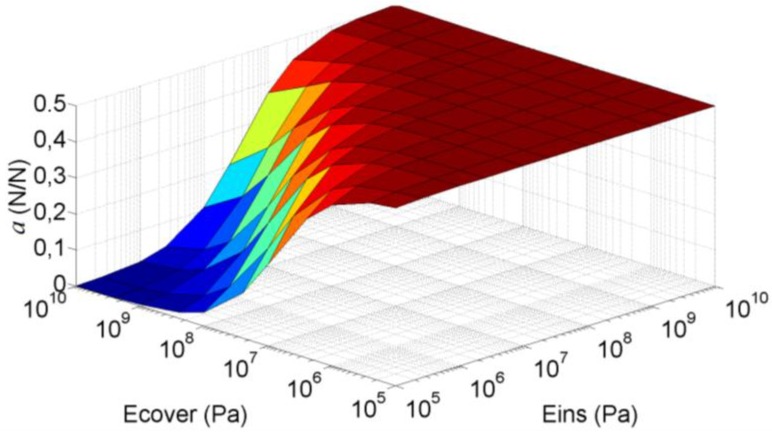
Parameter *a* as a function of the Young’s Modulus of the insulator and the cover calculated with the basic mechanical model.

Nevertheless, a balance of forces is also achieved if *K_ins_* ≫ *K*_cov*er*_ because we can neglect *K_ins_* in Equation (12) (we also assume *K_inn_* ≫ *K*_cov*er*_ and *K_out_* ≫ *K*_cov*er*_). Figure 6 shows how the parameter *a* changes for different values of the Young’s Modulus of the insulating and the cover for the sensor in Figure 1. The elastic constants of the other layers in Equation (13) are shown in Table 2 and the Young’s Modulus in Table 1. Note that *a* approaches 0.5, *i.e.* maximum sensitivity, for low values of the Young’s Modulus of the cover or for high values of the Young’s Modulus of the insulator. On the contrary, for increasing values of *E*_cov*er*_ and decreasing values of *E_ins_*, the parameter *a* and the sensitivity decrease. Note also that *a* ≤ 0.5 in Figure 6. Values of *a* above 0.5 would mean a negative value of *K_ins_* in the model of Figure 5, which obviously is not possible. Nevertheless, if the heights of the electrodes are not the same, values of *a* above 0.5 are possible, as will be discussed later.

**Table 2:** Elastic constants obtained from the Young’s modulus of the materials (see Table 1) and the geometry of the taxel (see Figure 1 and Equation (11)).

Layer	Elastic Constant *K* (N/m)
Substrate PEDOT	7.05 × 10^8^
Outer electrode	1.68 × 10^9^
Inner electrode	2.52 × 10^9^
Substrate	2.74 × 10^8^

### 4.3. Finite Element Analysis

The behaviour described in the previous section is confirmed by FEA simulations. Seven geometries have been defined in COMSOL for each simulation, six of them correspond to each layer of the sensor: the substrate, the outer electrode, the insulator, the inner electrode, the polymer ink on plastic and the cover. The seventh corresponds to an aluminium plate atop the sensor to exert the force through it. All the boundaries of these geometries have a free constraint condition except for the following:

1. The bottom boundary of the substrate has a fixed constraint condition.
2. The lateral boundaries of the substrate, the polymer ink on plastic layer and the cover have a symmetry plane constrain condition.
3. The top boundary of the aluminium plate has a free constraint condition and a load in the *y* axis direction.

**Figure 7:**
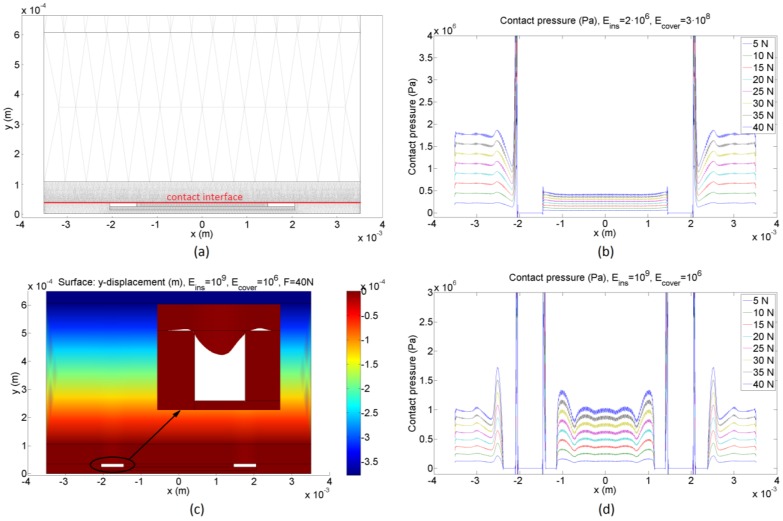
FEA simulations: (**a**) geometry and mesh, (**b**) example of contact pressure between the electrodes and the conductive polymer layer for low values of the Young’s Modulus of the insulator and high values of the Young’s Modulus of the cover, (**c**) example of indentation at the borders of the electrodes, and (**d**) example of contact pressure between the electrodes and the conductive polymer layer for high values of the Young’s Modulus of the insulator and low values of the Young’s Modulus of the cover.

Four identity boundary pairs were created to join the different boundaries of the geometries in contact with each other. Moreover, a contact pair was created to study the contact pressure distribution on each electrode surface. This contact pair is composed of the bottom boundary of the polymer ink on the plastic layer and the top boundary of the outer and inner electrodes. A default triangular mesh was used considering that the upper boundary of the contact pair has to have at least twice the number of nodes than the bottom one (see Figure 7a).

Figure 7 shows results from FEA simulations of the sensor in Figure 1. Low values of the Young’s Modulus of the insulator and high values of the Young’s Modulus of the cover cause unbalance between the forces on both electrodes as said in Section 4.2 and illustrated by Figure 7b. On the contrary, for high values of the Young’s Modulus of the insulator and/or low values of the Young’s Modulus of the cover, a balance in the forces at the interfaces of the inner and outer electrodes is observed, as shown in Figure 7d. Another remarkable feature that can be observed in Figure 7d is the effect of the indentation at the borders of the electrodes, which is especially noticeable in the case of soft covers. When the load is increased, the contact area in the borders is reduced theoretically to zero (see the inset in Figure 7c), so the pressure should be infinite in theory. Moreover, there is not contact between the conductive polymer and the electrode in the area close to the borders. The effect of this indentation is also observed in the uneven pressure profiles in Figure 7d.

**Figure 8:**
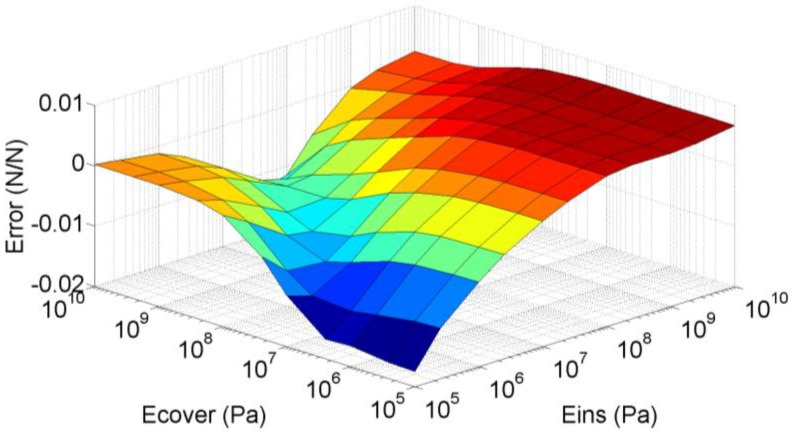
Difference between *a* calculated with the basic mechanical model and obtained from FEA simulations.

FEA simulations were carried out for Young’s Modulus of the insulator and the cover taking the values 10^5^ Pa, 2.5 × 10^5^ Pa, 10^6^ Pa, 2.5 × 10^6^ Pa, 10^7^ Pa, 2.5 × 10^7^ Pa, 10^8^ Pa, 2.5 × 10^8^ Pa, 10^9^ Pa, 2.5 × 10^9^ Pa and 10^10^ Pa. In this case, the force at the interface was obtained by integrating the pressure along the interface so the uneven profiles observed in Figure 7d are taken into account. The difference between the surface depicted in Figure 6 and that obtained from FEA simulations is shown in Figure 8. The maximum difference in *a* for these simulations is 3.5%FS. Moreover, from Equation (10):

(15) $$\frac{dS}{S}=\frac{1-2\cdot a}{1-a}\cdot \frac{da}{a}$$

so dS/S increases for decreasing values of *a*, and the maximum variation of the sensitivity is also 3.5%FS for a=0.

Therefore, there is a reasonable agreement between the sensitivity predicted by the simple model in Section 4.1 and Section 4.2 and the FEA simulations despite of the observed influence of the indentation. However, if there are significant deviations between the implemented sensor and this simple model, the sensitivity cannot be obtained only from Equations (10) and (13). This is covered by the next Section 4.4 and Section 4.5.

### 4.4. Limitations of the Simple Model

The simple model of Section 4.1 and Section 4.2 makes the following assumptions that also constitute goals of an ideal design:

1. The contacts are identical or at least the relationship between $\alpha_{inn}$ and $\alpha_{out}$ in Equation (6) does not depend on F.
2. The force is distributed between both electrodes as stated in Equation (7), *i.e.* the total force on the taxel equals the summation of the forces on the electrodes.
3. The balance between the forces on both electrodes, *i.e.* the parameter *a*, does not depend on F.
4. The electrodes are flat and are at the same height.
5. The pressure along the surface of the contacts is uniform.

**Figure 9:**
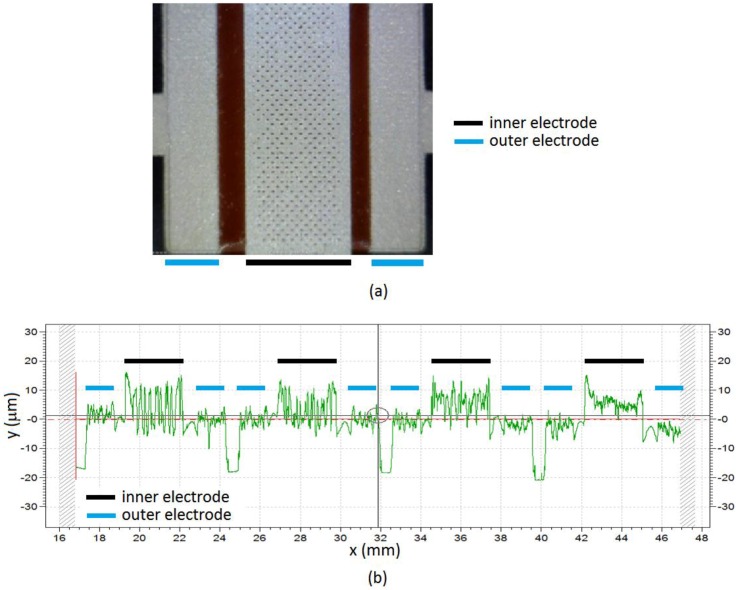
(**a**) Microphotograph of the sensor with redEL insulator layer, and (**b**) profilometry of one row of the sensor.

**Figure 10:**
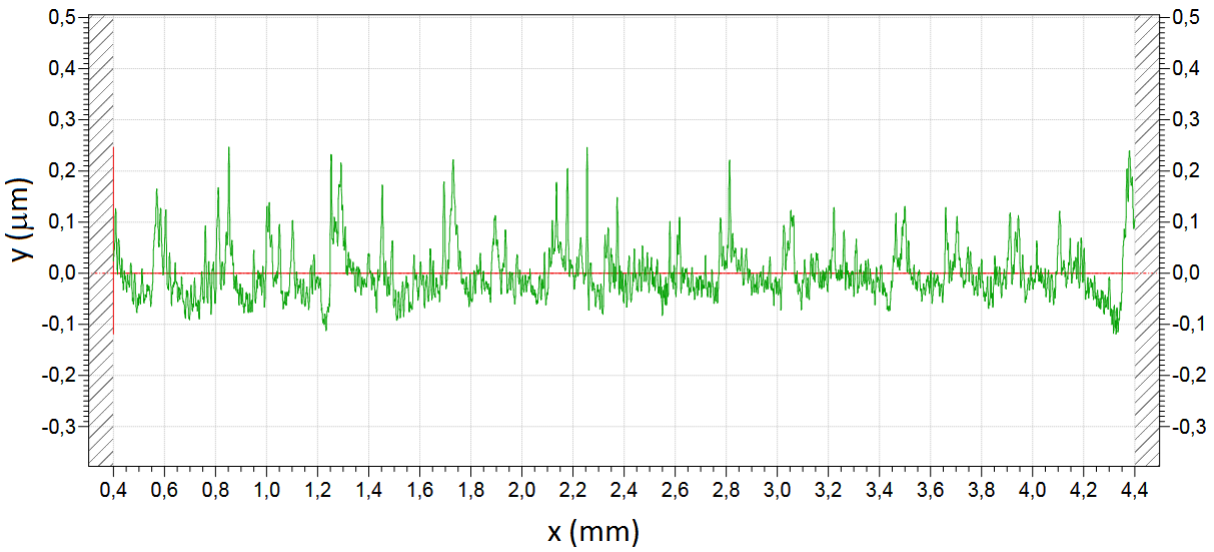
Profilometry of the sensitive side of the conductive polymer layer.

However, these assumptions are not valid for the sensors fabricated with the technology described in Section 2. The reason is that this technology achieves profiles of the sensor without the conductive layer such as those in Figure 9b, and the profile of the sensitive side of conductive polymer layer is shown in Figure 10. Both roughness, that of the electrodes and of the conductive polymer can be added to consider an equivalent interface between the resulting rough surfaces and a flat surface one [37]. We observe from these figures that they show a significant roughness. This is confirmed by the microphotograph in Figure 9a, where it is observed that the deposition of the silver layer on the insulator is not even, which is especially noticeable in the case of the redEL insulator. Moreover, the electrodes are at different heights, and peaks of insulator as high as, or even higher than, that of the inner electrode are observed in some profilometries, so Equation (7) is not fulfilled. Finally, the simulations in Section 4.3 show uneven pressure profiles along the electrodes in many cases and a noticeable increase of the pressure at the borders of the electrodes. The pressure profiles for the sensors with profilometries like that in Figure 9b are obviously much more complex.

**Figure 11:**
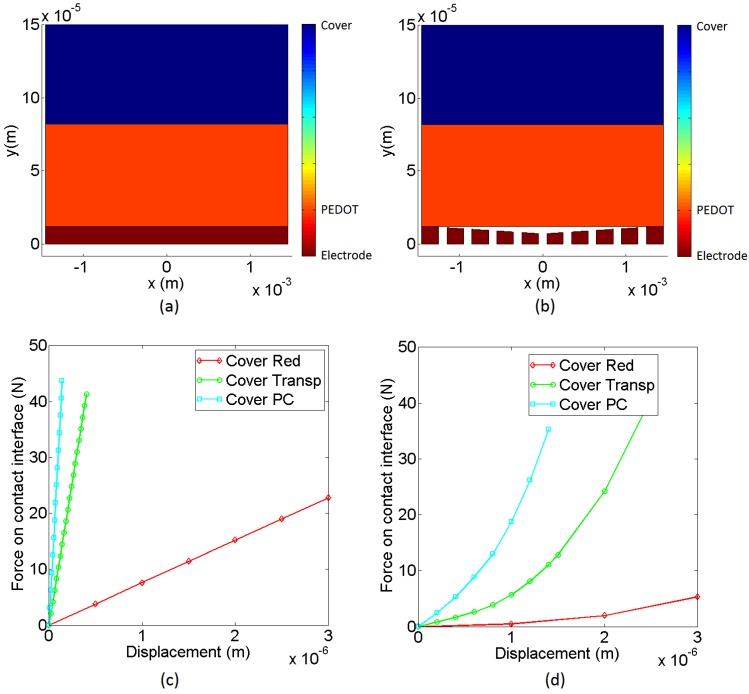
Geometries for the FEA simulation of (**a**) a flat contact and (**b**) a rough contact. Relationship between the force at the contact interface and the displacement of the cover obtained for (**c**) a flat contact and (**d**) a rough contact.

To illustrate the influence of roughness at the contact interface, Figure 11 shows a COMSOL simulation of a contact between a flat and a rough interface. The relationship between the force at the contact interface and the displacement of the cover is linear in the case of the flat electrode, while it is not for the rough interface. This can be modelled by a non-linear elastic constant, as explained in the following paragraphs.

The conductance at the contact interface can also be written as [33]:

(16) $$C_{x}=\frac{2}{\rho^{*}\cdot E^{*}}\cdot K_{\mathrm{int}}$$

where $\rho^{*}$ and $E^{*}$ are combined resistivity and elastic modulus of the materials in contact and $K_{\mathrm{int}}=dF_{x}/d\delta$ being $F_{x}$ the normal force on the contact and δ the displacement caused by it. This interfacial stiffness $K_{\mathrm{int}}$ can be considered in series with the other elastic constants in the model on Figure 5 [27]. In the thermodynamic limit [27] there is self-similarity and the statistical distribution of the microcontacts sizes and local pressures remains constant as the load increases, then $K_{\mathrm{int}}$ is linear with *F_x_* and Equation (3) is valid [26,27].

This is not fulfilled for small loads, when only a few microcontacts are established, nor when there are borders that cause indentation. Long wavelength features at the surface profile determine the behaviour in this regime, quantitatively through their amplitude and also qualitatively through their texture [38,39]. $K_{\mathrm{int}}$ scales sublinearly with *P* and a large difference in response is expected between samples. As the load increases, the contact area ‘allowed’ by long wavelength features reaches a maximum and the interfacial stiffness and the conductance scales again linearly with the pressure on the contact. Nevertheless, this only happens if this long wavelength is smaller when compared to the contact size [26]. If this is not fulfilled, the relationship is not linear and again the mismatching between samples is large. Therefore Equation (3) should be written as:

(17) $$C_{x}=k_{x}\cdot P_{x}^{m}$$

where *m* = 1 in the thermodynamic limit. For small or medium loads, there is a sublinear dependence, *m* < 1. Actually, *m* depends on the fractal dimension *D* (or on the Hurst exponent) of the surface profile [27,40]. For a finite squarish indenter [40] gives the value m≈0.2567⋅D that ranges from 0.51 to 0.77 for a fractal dimension from 2 to 3.

### 4.5. Extended Model

**Figure 12:**
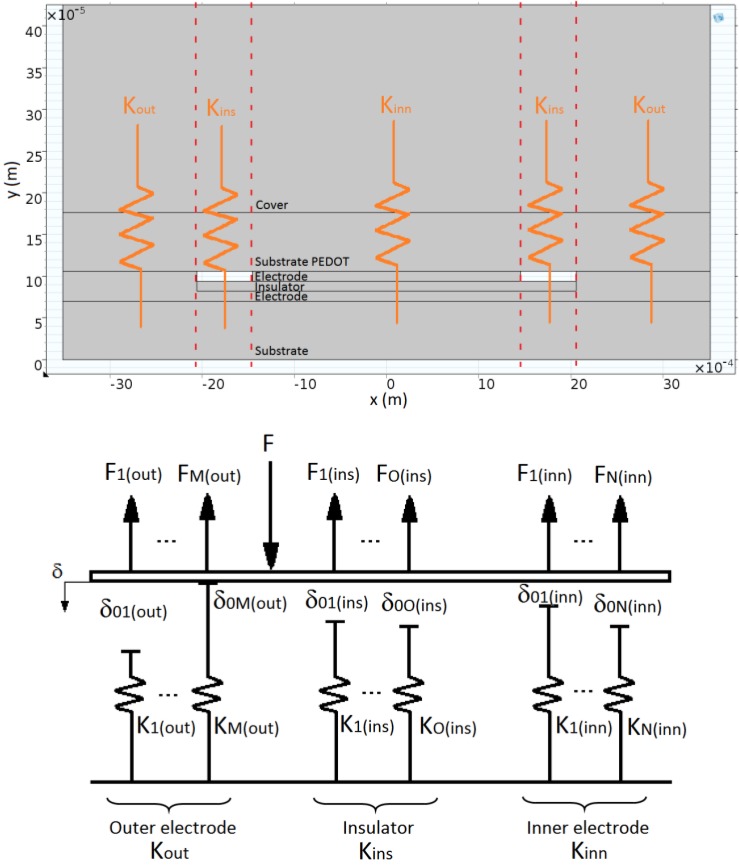
Winkler model of the taxel.

From the analysis in the previous section it is clear that long wavelength features of the surface profile have a direct impact on the sensor response, specifically in its sensitivity and linearity. Detailed simulation of the complete sensor in Figure 9 that includes fine roughness, is too complex and does not reach a solution. An alternative to explain the sensor response, regarding the above mentioned limitations, is the use of a Winkler foundation model. This simple model splits the elastic layer into beams with an associated elastic constant. It has been used in [41] to model the cover of the sensor in the fingers of a robotic hand. The model can be extended to multiple layers [42]. A model of the taxel with profilometries like that in Figure 9b can be made in this way by a set of independent beams, each one composed of different layers, as Figure 12 depicts. To model long wavelength roughness features, the beams of the model are placed at different heights, so the contact is first established with the beams at a highest height and the others will make contact gradually as the load increases. Note that this approach resembles the classical theories in the sense that contacts at beams intend to model highly clustered micro-contacts that act as a single contact equal to the envelope size [25], the position of the clusters being determined by the large-scale waviness of the surface, and the micro-contacts by the small-scale surface roughness [25,33,43]. Moreover, [34] predicts a linear relationship between the electrical conductance and the normal force regardless of the assumed shape of the asperities at the contact interface. However, these models lack the inclusion of interaction effects between asperities [44]. These effects are contemplated in [45] by introducing not only a local but a global displacement of the asperities in contact. Our approach resembles this model in the sense that the force exerted by one beam depends on the force exerted by the others as:

(18) $$F_{i}=\{\begin{array}{cc} K_{i}\cdot (\delta -\delta_{0i}) & \delta \geq \delta_{0i}\\ 0 & \delta <\delta_{0i} \end{array}$$

(19) $$\sum_{i}^{N}K_{i}\cdot (\delta -\delta_{0i})=F;\;\forall i\;such\;that\;\delta \geq \delta_{0i}$$

(20) $$\delta =\frac{F+\sum_{i}^{N}\left(K_{i}\cdot \delta_{0i}\right)}{\sum_{i}^{N}K_{i}};\;\forall i\;such\;that\;\delta \geq \delta_{0i}$$

Note that $F_{i}$ depends on δ, and δ is determined by all the beams in contact, *i.e.* $F_{i}$ has to be calculated iteratively. If the area of all beams is the same:

(21) $$F_{inn}=\sum_{i=1}^{N}F_{i(inn)}\;F_{out}=\sum_{i=1}^{M}F_{i(out)}\;F_{ins}=\sum_{i=1}^{O}F_{i(ins)}$$

where N, M and O are the number of beams to model the inner electrode, outer electrode and the insulator respectively. The normal force on the taxel is now:

(22) $$F=F_{inn}+F_{out}+F_{ins}$$

so Equation (7) does not fulfil because part of the force is borne by the insulator.

Moreover, taking into account the more general expression in Equation (21), Equation (6) can be rewritten as (note that the contact is always between PEDOT and silver, both in the inner and in the outer electrode):

(23) $$V_{out}=\alpha \cdot \frac{\sum_{i}^{N}F_{i(inn)}^{m}\cdot \sum_{i}^{M}F_{i(\mathrm{out})}^{m}}{\sum_{i}^{N}F_{i(inn)}^{m}+\sum_{i}^{M}F_{i(\mathrm{out})}^{m}}\cdot R_{gain}\cdot V_{bias}$$

This extended model, though still simple, contemplates different sizes of the electrodes, different forces on the electrodes and different heights of the electrodes. The latter is implicit in the model and introduces the possibility that there is a threshold due to the absence of contact with one of the electrodes. The progressive settling of contacts with the beams in Figure 12 also introduces the effect of texture and the dependence of the balance of force between the electrodes on the total force on the taxel *F*. Finally, the parameter *m* contemplates the nonlinearity when the thermodynamic limit is not reached, for instance for low loads. Taking into account that the contact is always settled between the conductive polymer PEDOT and the silver electrodes, we assume the same fractal dimension for the contact at a beam regardless of the insulator or the cover that are used. For the sake of simplicity we do not introduce the dependence of *m* on the load ([27] provides a complex theoretical expression for the limit between the sublinear and linear regimes but we do not have all the data to contemplate it in the model). In summary, the model overcomes many limitations of the simpler previous one described in Section 4.1 and Section 4.2.

## 5. Experimental Results and Discussion

Two sets of experiments were carried out to explore the performance of different realizations of the sensor. A first set was devoted to see the influence of the insulator. Five sensors with different insulators were fabricated as explained in Section 2. The set-up in Figure 3 was used to exert a normal force on the taxel. A square 15.24 mm side and 4 mm thick metal piece was placed between the force sensor probe and the taxel under test.

**Figure 13:**
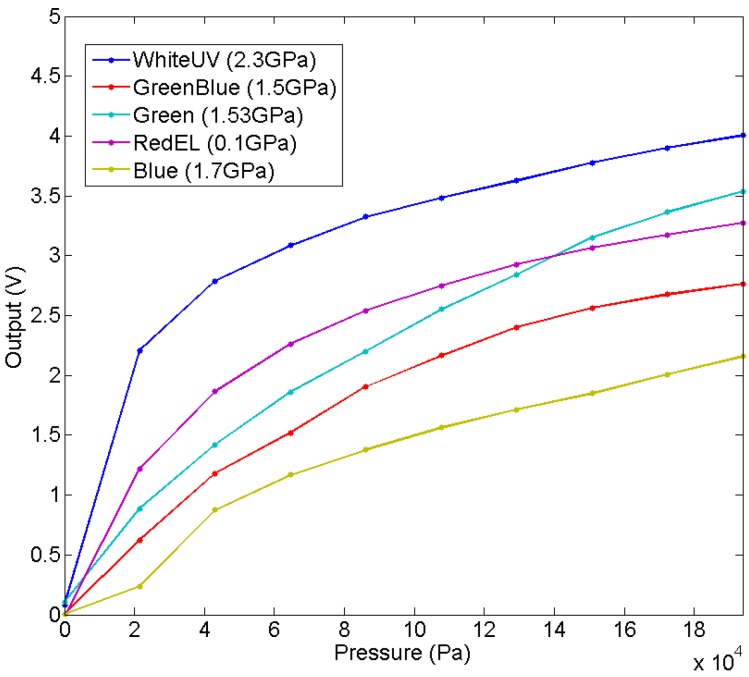
Average of the output of the taxels for sensors with different insulator layer.

**Figure 14:**
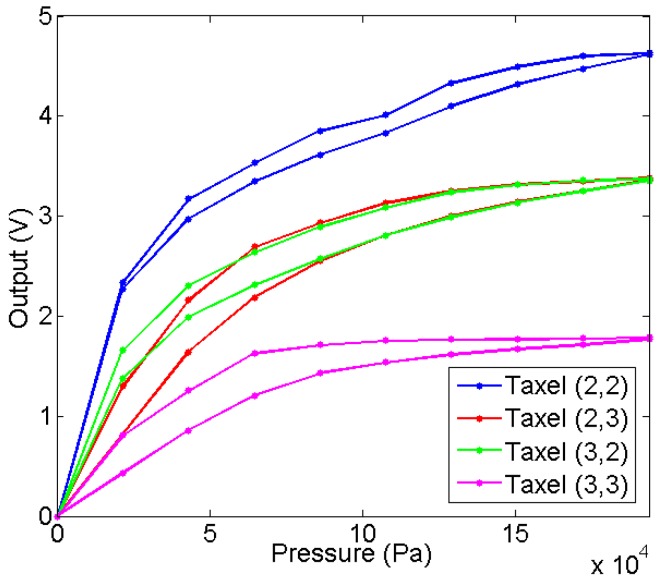
Output of central taxels for the sensor with redEL insulator.

Figure 13 shows the average of the output of the taxels for five tactile sensors with different insulators. The comparison of these curves clearly shows that Young’s Modulus does not determine the sensitivity of the sensor. Moreover, this sensitivity also depends on the pressure on the tactile sensor because the curves are not linear. The explanation is that the roughness at the contact interface between the electrodes and the condutive polymer has a large influence for this size of the taxel, because it is high in comparison to the size of the taxel, so a large mismatching between taxels is expected (see Section 4.4). To illustrate it, Figure 14 shows the output of the four central taxels of the sensor with redEL insulator, note that there is a large difference between the curves.

**Figure 15:**
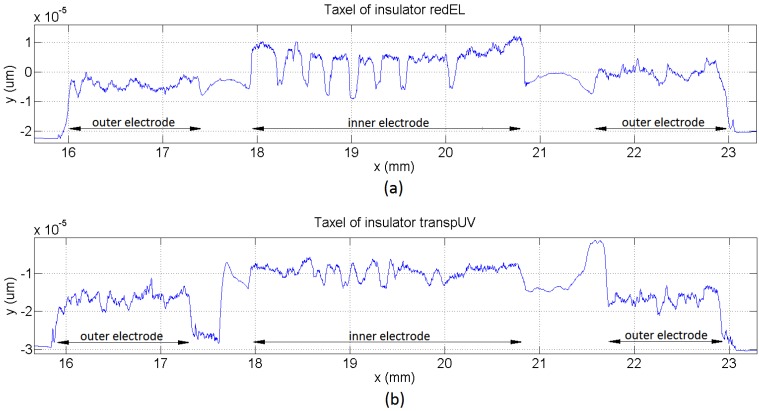
Profilometry of a taxel with (**a**) redEL insulator and (**b**) transpUV insulator.

**Figure 16:**
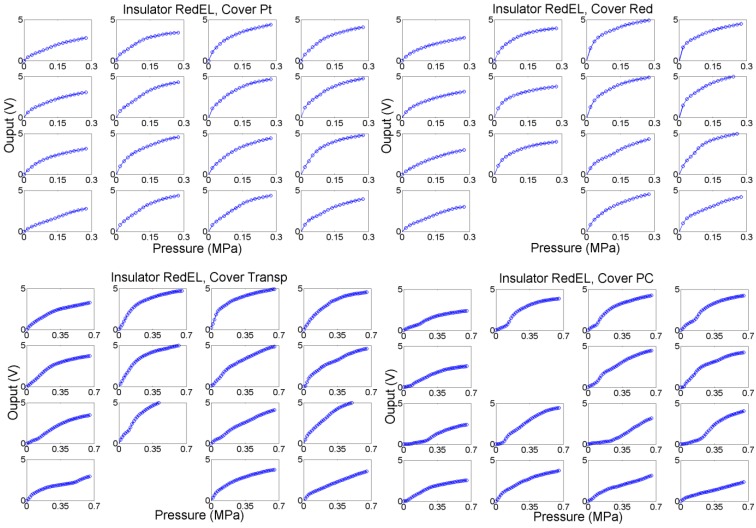
Output of taxels for the sensor with redEL insulator layer and different cover layers.

**Figure 17:**
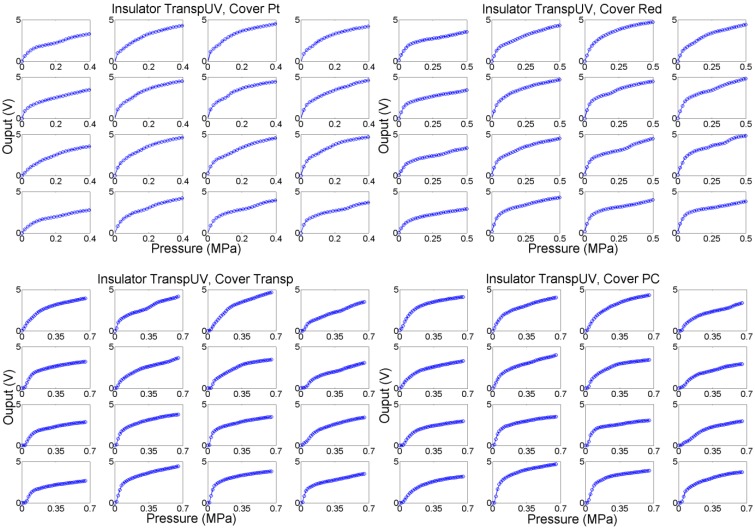
Output of taxels for the sensor with transpUV insulator layer and different cover layers.

A second set of tests was carried out with two insulators, the transpUV one (E = 3.3 GPa) and the redEL one (E = 0.1 GPa) with high and low Young’s Modulus respectively. The profilometries of a sample taxel of the sensors with both insulators are shown in Figure 15. This time the output was also registered for four different covers (see Table 1). Moreover, the metal piece placed between the cover and the force sensor was 7.62 mm side and 4 mm thick, and the outputs of all the taxels were registered. Figure 16 shows the outputs of the taxels of the 4 × 4 tactile sensor with the redEL insulator while Figure 17 depicts the output of the taxels of sensors with the transpUV insulator (the output of a few taxels that did not work properly has been removed).

The discussion and models in Section 4 can be used to understand the response of the sensors in Figure 16 and Figure 17. We can use the profilometry of a taxel from the sensor with redEL insulator in Figure 15a to build a model as stated in Section 4.5. We observe from this profilometry that there is a quite clear u-shape envelope in the inner electrode. We also can see that there are deep valleys where the contact of this electrode with the sensitive layer is unlikely. To model the latter feature, we set the condition that a twenty percent of the beams never make contact. To model the u-shape envelope a v-shape profile is used as envelope of the beams. Moreover, the slope of the two pieces of the v-shape of different taxels follows a normal distribution (with the valley point between an interval of 15 microns). The heights of the beams of the model are then obtained by adding the height of the envelope to another term, also dependent of a normal distribution (6⋅σ=3 microns). Finally, a normal distribution of the difference between the average heights of the beams of both electrodes was also introduced (6⋅σ=5 microns).

**Figure 18:**
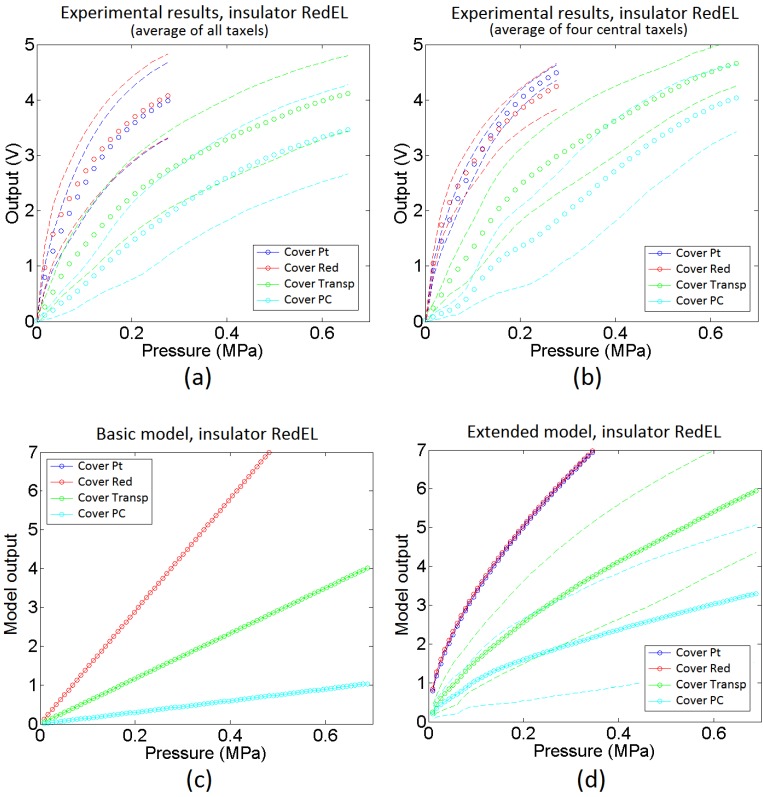
Output for a sensor with redEL insulator: (**a**) average of the output of all taxels, (**b**) average of the output of the four central taxels, (**c**) output of the basic model, and (**d**) output of the extended model.

The average of the curves in Figure 16 are shown in Figure 18a with circles of different colours associated to the four covers. The dashed lines in this figure are the boundaries of the range of variation of the averaged curves. Figure 18b shows the average of the four central taxels to isolate the border effects. Moreover, Figure 18c shows the output of the ideal model in the Section 4.2 and Figure 18d shows the average of sixteen curves given by the extended model in the Section 4.5.

**Figure 19:**
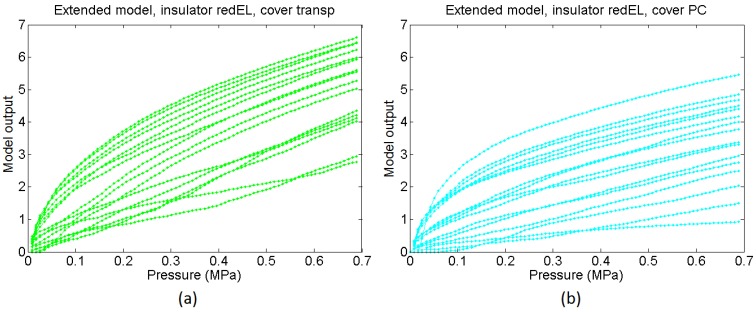
Set of sixteen output curves from the extended model for a sensor with redEL insulator and (**a**) transp cover and (**b**) PC cover.

From the comparison of the average curves in Figure 18a and the output of the basic model in Figure 18c it can be concluded that the basic model is able to predict quite well the dependence of the sensitivity on the cover. However, the average of the experimental curves is not as linear as the output from the basic model. The extended model provides a response closer to the experimental one in this sense, and its output curves are slightly sublinear. Nevertheless, we are comparing average curves from sixteen taxels, so actually the comparison is somewhat equivalent to that made for taxels sixteen times larger than that in Figure 1, because random features are partially filtered. This can be useful to predict the behaviour of a sensor with lower spatial resolution made using the same technology, but Figure 16 shows curves that diverge quantitatively and also qualitatively from the average output. The extended model with random parameters is able to reproduce such behaviour. Specifically, the mismatching of the curves associated to soft covers such as the Pt and the Red is quite low, as observed in Figure 18b, where the dashed lines are close to the average curve. On the other hand, the mismatching between the response of different taxels of the sensor is quite large for rigid covers (labelled Transp and PC in the figures). This is also observed in the output of the extended model, where the dashed lines are far from the averaged curves for these covers. Moreover, this variation is not only quantitative, *i.e.* it is not only a change in the sensitivity of the curve, but the shape of the curves varies quite significantly from one taxel to the other. As stated in Section 4.5, the reason is the progressive settling of contacts with the beams in the model or the asperities in the real contact interfaces. Figures 19a,b show sixteen sample curves obtained from the extended model for low compliance covers (Transp and PC), and they also have quite different shapes, some of them even showing a few knee points where the slope changes.

Regarding the sensor made with the low compliance insulator transpUV, its profilometry is shown in Figure 15b. The profile of the external electrodes is similar to that in the sensors with the redEL insulator (Figure 15a), which is logical because they are made on the same substrate in both cases. However, the profile of the inner electrode is different. Firstly, there is not a clear envelope as in the sensor with the redEL insulator (the u-shape). Secondly, the shape of the profile is somewhat similar to a triangular waveform, while it is closer to a square waveform (added to the envelope) in the profilometry of the redEL insulator in Figure 15a. Moreover, although the valleys in Figure 15b are not as deep as in Figure 15a, they are also present in the profile of the inner electrode in Figure 15b. Finally, it is worth noting that the insulator has a remarkable uneven profile in the gap between both electrodes in Figure 15b, with peaks higher than those of the internal electrode.

**Figure 20:**
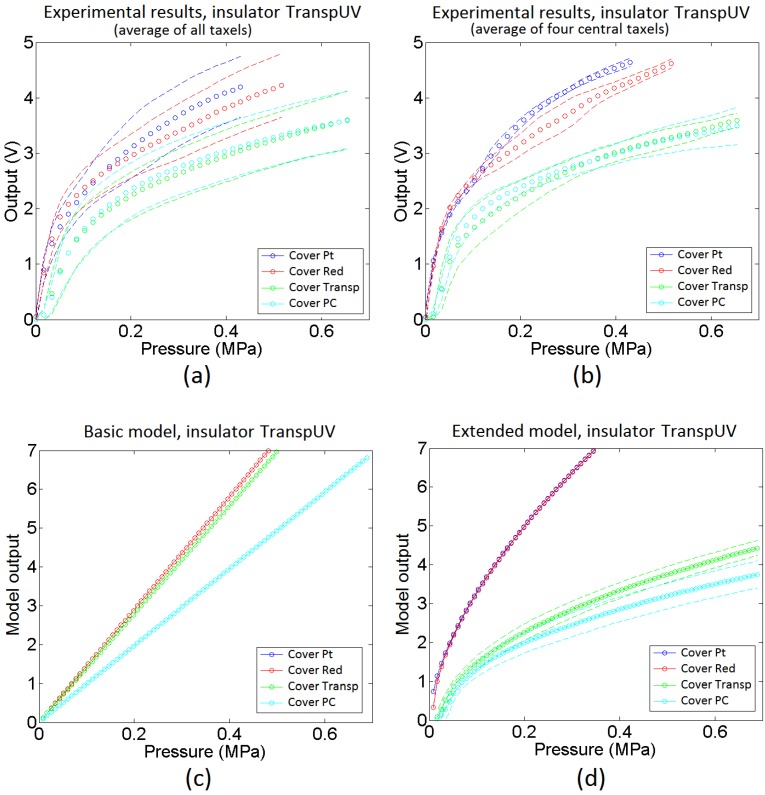
Output for a sensor with transpUV insulator: (**a**) average of the output of all taxels, (**b**) average of the output of the four central taxels, (**c**) output of the basic model, and (**d**) output of the extended model.

**Figure 21:**
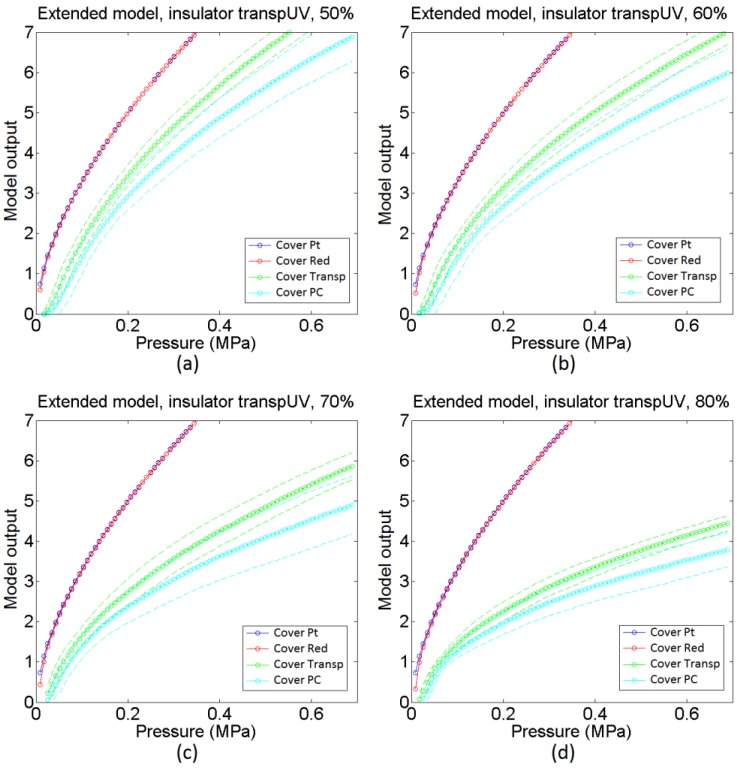
Output from extended model with transpUV insulator where different percentages of the total number of beams were removed: (**a**) 50%, (**b**) 60%, (**c**) 70% and (**d**) 80%.

**Figure 22:**
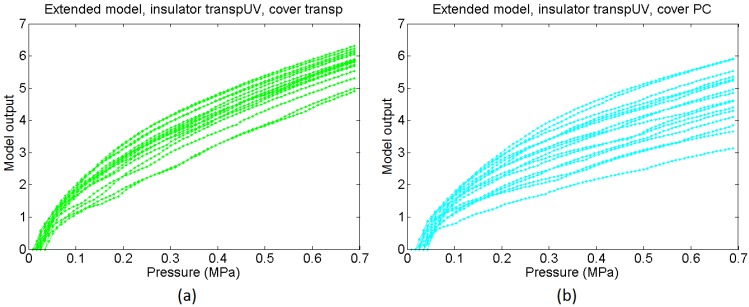
Set of sixteen output curves from the extended model for a sensor with transpUV insulator and (**a**) transp cover and (**b**) PC cover.

Figure 20 shows the same information that Figure 18 but for the sensors made with the transpUV (low compliance) insulator. The output of the basic model in Figure 20c does not reflect well this time the dependence of the sensitivity with the cover in the case of low compliance covers (Transp and PC). With respect to the extended model, as a first approach the heights of the beams in Figure 12 were generated from a random normal distribution (6·*σ* = 5 microns), without adding any envelope term. However, the output of the obtained model did not resemble the experimental data in Figure 20a. The curves provided by the model were more linear and had more sensitivity than the experimental curves. The reason for this difference is the limitation of the Winkler model to contemplate the interaction between beams. This interaction is clear when both, the insulator and the cover, are made of low compliance materials. As said above, the profile of the inner electrode in Figure 15b shows peaks that are more shapely than those in the sensor with the red insulator (they are quite flat in Figure 15a). As a consequence, taking into account the interaction between beams, the actual contact area is a small percentage of the area of the electrode. If this effect is introduced in the model by setting a number of beams that never come into contact with the sensitive layer, a knee point appears in the average output curve provided by the model. This is illustrated in Figure 21, where different percentages of the total number of beams were removed. It can be seen that the higher this percentage the higher the change of the slope or second derivative in the knee point, so the previous interpretation is confirmed. Nevertheless, once this condition is set and the beams are removed, the response of the model is similar to the experimental data in Figure 20b. Specifically, it predicts a higher sensitivity and low mismatching for soft covers (Pt and Red), and lower sensitivity, less linearity and larger mismatching for low compliance covers (Transp and PC). It also reflects the existence of a small threshold that can be seen for instance in the curves in Figure 17 for low compliance covers, and is due to the lack of contact with one of the electrodes. Finally, the mismatching between the curves provided by the model for the transpUV insulator is now lower than that observed for the redEL insulator and the same covers in Figure 18b, which is also observed in the experimental data. This is also confirmed by Figure 22, that shows the result of a similar simulation to that displayed at Figure 19 but for the sensors with transpUV (low compliance) insulator. Note that the dispersion or mismatching between curves is lower in Figure 22 than in Figure 19.

**Figure 23:**
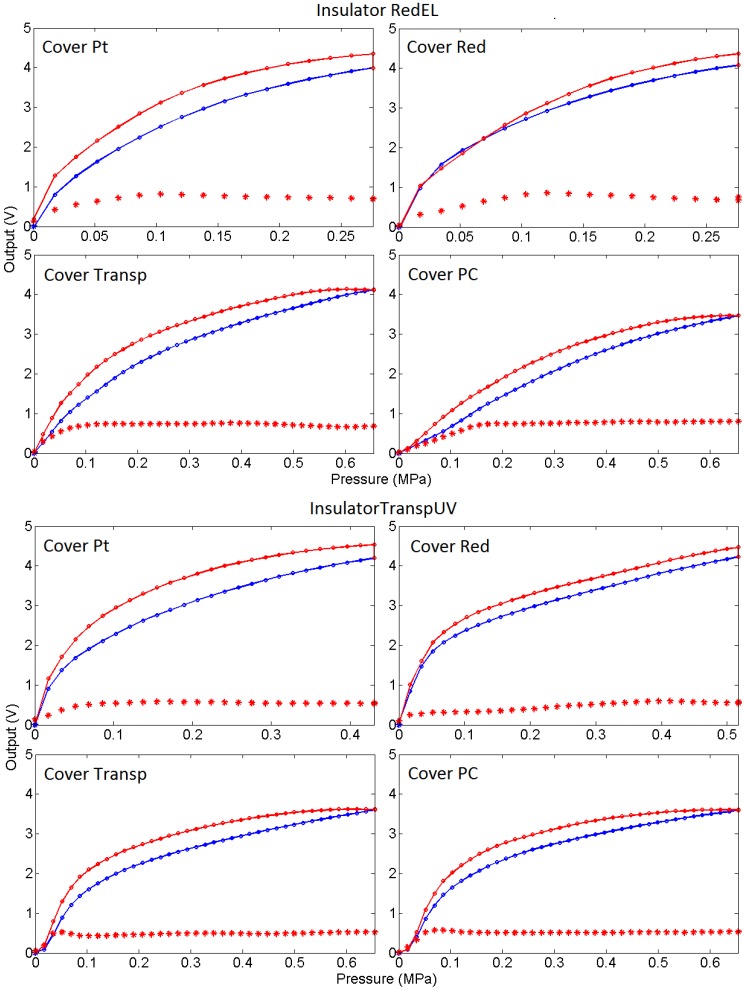
Average of the loading-unloading curves (blue and red curves respectively) of the sensors with different covers and insulator layers. Asterisks represent the standard deviation of the curves.

To complete the analysis of the behaviour of the sensors, Figure 23 shows the average of the loading-unloading curves of the sensors with different covers, and Table 3 shows data related to the hysteresis measured in the average output curve, and also the area below the absolute value of the second derivative of the loading curve, the last one used as a figure to understand how linear the curve is. Note that the cover has also a significant influence on the hysteresis, the Red being that with less hysteresis. Regarding the linearity, the average curves in Figure 23 and the data of the area under the second derivative curve in point to a better behaviour of the sensors with the soft redEL insulator. However, these are average values and there is a large mismatching between the curves from different taxels for low compliance covers, as said above. Generally speaking, the linearity is improved with soft covers in the whole input range, while a knee point is observed in the curves from the sensors with low compliance insulator and cover. However, the curve is quite linear to the right of this knee point, though the sensitivity is lower.

**Table 3:** Parameters of the curves.

Insulator	Cover	Hysteresis (%)	Area 2nd derivative (V/MPa)
RedEL	Pt	14.03	36.90
	Red	7.35	45.59
	Tranps	14.87	22.96
	PC	13.58	24.40
TranspUV	Pt	15.27	49.66
	Red	7.60	45.88
	Transp	13.58	51.59
	PC	11.68	48.01

## 6. Conclusions

This paper analyses the response of sensors made with a common approach and a low cost technology. The discussions and conclusions can be valid for other sensors that exploit the same principle. A clear discrepancy between the experimental data and the basic model that assumes an ideal taxel is observed. This ideal model provides a linear output whose slope can be tuned by changing the compliance of the insulator between conductive tracks or of the cover atop the sensor. However, the response of the sensor diverges from this behaviour except in the case of a soft insulator and for the average of the curves from all taxels. Nevertheless, the inspection of these curves from different taxels show a large mismatching for low compliance covers. They differ quantitatively (different sensitivity) and also qualitatively (different shape). The mismatching is lower for low compliance insulators and covers, but the curves have a noticeable knee, though they are quite linear for increasing input values.

The explanation of all these main observed features requires a finer modelling, since the profilometries show that the real electrodes are different and are at different heights, the insulator has peaks at similar or even higher height that the electrodes and there is a remarkable roughness at the contact interface. Some results from studies of the physics of contact between rough surfaces are incorporated. Specifically, a Winkler model is implemented to contemplate the roughness. The taxel is split into many beams composed by slices of the layers that form the taxel. The conductance associated to each beam depends on the force it supports. The conductance of the beams of the same electrode is aggregated, and the resulting conductance from both electrodes is computed by a parallel operator. The result is not linear if the relationship between the conductance of both electrodes depends on the force. The profilometry of the model is inspired in the measured one, with a few random parameters. This model explains quite well the experimental data from a sensor with soft insulator. However, it does not fit the behaviour of the sensor with a low compliance insulator because the Winkler model does not contemplate the interaction between beams. After a heuristic and simple change, this interaction is introduced and the result confirms that the knee in the curve is mainly explained by an actual contact area of the inner electrode much smaller than its total area. In short, the differences of the contact interface of both electrodes cause a nonlinear response. This nonlinearity is also explained by the progressive making of contact with the beams when the load increases. Moreover, FEA simulations also show a clear effect of the indentation at the borders. Covers of soft materials can be used to improve the result and have a response closer to the ideal model, though the error due to border effects is larger. A sensor with taxel of larger area also performs better, as seen from the average curves of the sensors in the paper. A technology that achieves more uniform contact profiles, where the roughness shows a wavelength much shorter than the size of the contact, will also perform better. The borders at the mechanical contact interface should be minimized, being the ideal contact interface a flat one along the whole sensor area with the mentioned fine roughness added. Finally, from the results and discussions of the paper, it is envisaged that a taxel with only one active contact interface would provide better results.
